# Transcriptome Analysis of Gene Expression Patterns Potentially Associated with Premature Senescence in *Nicotiana tabacum* L.

**DOI:** 10.3390/molecules23112856

**Published:** 2018-11-02

**Authors:** Zhe Zhao, Yifan Li, Songchao Zhao, Jiawen Zhang, Hong Zhang, Bo Fu, Fan He, Mingqin Zhao, Pengfei Liu

**Affiliations:** College of Tobacco Science, Henan Agricultural University, Zhengzhou 450002, China; ycxyzz@126.com (Z.Z.); liyifan81@163.com (Y.L.); zhaosongchao1314@126.com (S.Z.); zjw960212@163.com (J.Z.); today.hong@163.com (H.Z.); fubo0527@126.com (B.F.); hefan.87@163.com (F.H.); liupengfei523@126.com (P.L.)

**Keywords:** premature senescence, tobacco, transcriptome, ultrastructure, plant hormone

## Abstract

Senescence affects the remobilization of nutrients and adaption of the plant to the environment. Combined stresses can result in premature senescence in plants which exist in the field. In this study, transcriptomic analysis was performed on mature leaves and leaves in three stages of premature senescence to understand the molecular mechanism. With progressive premature senescence, a declining chlorophyll (chl) content and an increasing malonaldehyde (MDA) content were observed, while plasmolysis and cell nucleus pyknosis occurred, mitochondria melted, thylakoid lamellae were dilated, starch grains in chloroplast decreased, and osmiophilic granules increased gradually. Moreover, in total 69 common differentially expressed genes (DEGs) in three stages of premature senescing leaves were found, which were significantly enriched in summarized Gene Ontology (GO) terms of membrane-bounded organelle, regulation of cellular component synthesis and metabolic and biosynthetic processes. The Kyoto Encyclopedia of Genes and Genomes (KEGG) pathway analysis suggested that the plant hormone signal transduction pathway was significantly enriched. The common DEGs and four senescence-related pathways, including plant hormone signal transduction, porphyrin and chlorophyll metabolism, carotenoid biosynthesis, and regulation of autophagy were selected to be discussed further. This work aimed to provide potential genes signaling and modulating premature senescence as well as the possible dynamic network of gene expression patterns for further study.

## 1. Introduction

Senescence is the final phase of plant development, it promotes the remobilization of nutrients [1] and makes plants adapt well to the environment [2]. As a regulated degeneration process, leaf senescence undergoes a series of degradations in cell structure and gene expression [3,4]. The chlorophyll content, photosynthetic capacity decreases [5], and the ROS levels [6] and phytohormones changed, along with the degradation of chloroplast, mitochondria, and nucleus [7] during senescence. 

According to previous reports, many environmental stress conditions, such as dark [8], heat [9], light [10] and so on, as well as some endogenous factors [2,11], trigger premature senescence. Nowadays, with the process of global warming, the environment is becoming even tougher for life on the earth due to the negative effects of high temperature [12]. Environmental conditions may affect plant growth and even induce premature senescence, thus leading to the decrease of plant yield and quality [13,14,15]. As reported by Ron Mittler [16], the most lethal stress condition for plants in the field, is the combination of several simultaneous abiotic stresses rather than a particular stress condition, and the tolerance to combined stresses could be a research focus. Therefore, studying the molecular mechanism underlying premature senescence lays the important foundation for the basic research of senescence and for the better response to stress conditions.

It is well known that plant hormones play crucial roles in regulating senescence. Parts of hormones, such as abscisic acid (ABA), ethylene (ET), salicylic acid (SA), and jasmonic acid (JA), have been shown to regulate the plant response to stresses and accelerate leaf senescence [17,18], while the other hormones, including indoleacetic acid (IAA), cytokinin (CK), and gibberellin (GA), may positively participate in plant senescence [6]. Recently, Dek reported that there was a decrease in cytokinins and an increase in ABA at postharvest leaves, nevertheless increasing ABA concentration could suppress postharvest senescence [19]. Phosphatidylinositol 3-kinase (PI3K), a key enzyme of phosphorylating phosphatidylinositol, induced senescence by enhancing ethylene biosynthesis and signaling [20]. The mitochondrial AAA-protease gene *FtSH4* could control the SA synthesis and signaling in senescence via modifying the ROS level and *WRKY* transcription factors [21]. Furthermore, JA-regulated plant growth, senescence and defense involved many genes, including *ORA47*, *MYB59*, *MYC5* and so on [22,23]. Exogenous application of GA3 suppressed cabbage senescence, with an increasing expression of GA synthesis genes [24]. 

In addition, ROS production is the response of plants towards stresses [25]. It can induce senescence [26], and the elevated level of ROS could enhance the expression of senescence-associated genes (SAGs) [27]. A WRKY gene *BnaWGR* positively regulated leaf senescence, through modulating the expression of *RbohD* and *RbohF* genes, which encode the key enzymes related to ROS production [28]. Also, the expression of a plastid-targeted flavodoxin could function in mitigating senescence symptoms by preventing ROS formation in chloroplasts [29]. 

Also, the mechanism of senescence involves many other pathways, such as chlorophyll metabolism, carotenoid biosynthesis, regulation of autophagy and so on. As a visible sign of senescence, the leaf yellowing phenotype was mostly determined by chlorophyll, which influences the photosynthetic efficiency [30]. Some chlorophyll degradation-related genes (CDGs) were upregulated to accelerate the senescence induced by stresses and exogenous factors [31,32,33], Carotenoids serve as pigments, membrane stabilizers, and precursors of some plant hormones, functioning significantly in plant growth and response to abiotic stress [34,35]. It has been demonstrated that the silencing of *SlIPT4*, which is encoding an isopentenyltransferase, resulted in the acceleration of leaf senescence [36]. Autophagy, which contributes to the remobilization of damaged cells interrupted by ROS [37], plays an important role in ageing senescence and stress-induced premature senescence [37,38]. Zhou identified 30 autophagy-related genes (ATGs), and demonstrated their manifold functions in response to environmental stresses [39]. Though the mechanism of premature senescence has been widely studied, it is still largely elusive [40].

Common tobacco (*Nicotiana tabacum*) is one of the economic crops cultivated worldwide [41], and is always regarded as a model organism on which to learn fundamental biological processes [42]. The genome sequence of tobacco was first obtained in 2014 [43], and awaits more research on molecular function. Based on its high-efficient transformation and regeneration, tobacco can serve as a suitable model to study senescence [44,45]. With the rapid development of the RNA-seq technique for acquiring mRNA transcriptome profiling to investigate the probable function of genes, most Senescence-Associated Genes (SAGs) have been able to be characterized by such a technique. However, the transcriptome analyses of plant senescence has been operated only on a limited number of plants, including *Arabidopsis* [46], wheat [1], cotton [7,17], common tobacco [41], sorghum [47], maize [48], and petunia corolla [49], and more plants are required. Though the only transcriptome analysis of senescence in *Nicotiana tabacum* was reported by Li [41], it has laid focus on the metabolites of nutrient remobilization rather than the gene transcription regulating senescence. Also, the research on premature senescence of plants under combination of several simultaneous abiotic stresses is still absent. 

In this study, the RNA-seq and transmission electron microscopy were performed on samples of mature and premature senescent tobacco leaves which were collected at the same time. Based on specific data analysis, some candidate genes were identified as being significantly associated with stress-induced premature senescence, while the dynamic image of gene expression patterns from maturity to severe premature senescence were revealed. This work, which served as the first reference of transcriptome analysis for combined stress-induced premature senescence, draws part of the holistic picture for plant developmental and premature senescence, enables us to understand the regulation mechanisms and functional categories of genes in premature senescence, and provides the resources for specific marker genes related to tobacco senescence.

## 2. Results

### 2.1. Phenotypic Characterization and Biochemical Analysis

**Figure 1:**
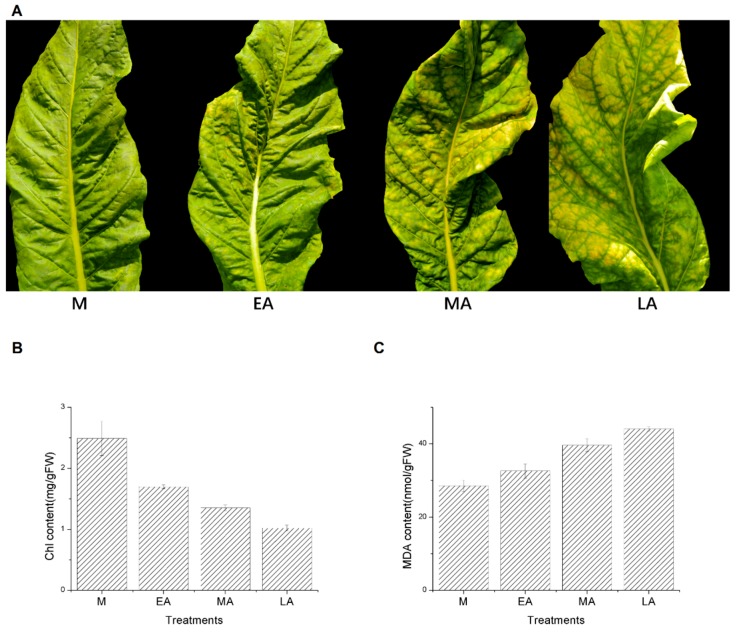
Plant phenotypes, Chlorophyll (chl) and malonaldehyde (MDA) measurements at the four stages of premature senescence. M, mature, EA early senescence, MA middle senescence, LA late senescence. (**A**) Appearance of tobacco leaves at four stages of premature senescence. (**B**) chl content. (**C**) MDA content. Error bars indicate the means ± SD (n = 3).

In this study, the four stages were chosen according to the visible symptom of leaf yellowing rate to judge the degree of senescence [47,50,51,52]. Leaves which had been green and fully expanded were defined as Mature (M), and the other leaves of Early Senescence (EA), Middle Senescence (MA), Late Senescence (LA) could be collected according to the yellowing rates of about 10%, 25%, and 50% (Figure 1A). Then the significant declining chl content and increasing MDA content showed the same tendency for leaves senescence (Figure 1B,C), proving that the leaves for RNA-seq to identify premature senescence were credible.

### 2.2. Ultrastructural Changes in Senescing Leaves

**Figure 2:**
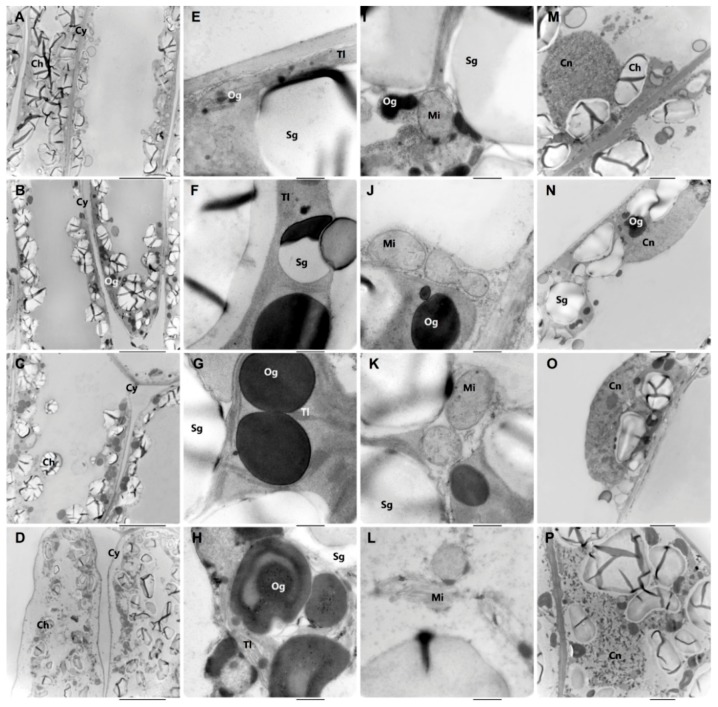
Ultrastructure of mesophyll cell at four stages. (**A**,**E**,**I**,**M**) Leaves of mature stage (**M**). (**B**,**F**,**J**,**N**) Leaves of early premature senescence stage (EA). (**C**,**G**,**K**,**O**) Leaves of middle premature senescence stage (MA). (**D**,**H**,**L**,**P**) Leaves of late premature senescence stage (LA). **A**–**D**, scale bars in bottom right corner of every picture correspond to 10 μm; **E**–**L**, scale bars correspond to 500 nm; **L**–**P**, scale bars correspond to 2 μm. Sg, starch grain; Tl, thylakoid lamellae; Og, Osmiophilic granules; Ch, Chloroplast; Cn, Cell Nucleus; Mi, Mitochondria; Cy, cytoderm.

The changes of cell ultrastructure during senescence were analyzed using Transmission electron microscopy (Figure 2) and shown in Table 1. At the first senescence period (M), the cell structure exhibited the normal status, for the mitochondria, while chloroplast and cell nucleus were regularly ordered. The starch grains in the chloroplast were large and numerous, and there were few osmiophilic granules in the chloroplast (Figure 2E,I). When leaves began to be senescent, plasmolysis of the cell occurred gradually, and the degree of plasmolysis increased with the senescence procedure, from M to LA (Figure 2A–D). Also, the starch grains decreased in quantities and sizes, with the size and number of osmiophilic granules in the chloroplast increasing. The size of osmiophilic granules increased sharply from M to EA, as well as the amount (Figure 2E–H). As for the thylakoid lamellae in the chloroplast, regular arrangements were found in M, EA, and MA, in which the amount of lamellae increased, while the lamellae in LA were multiple, disordered, and severely dilated (Figure 2I–L). It was obvious that the mitochondria became lightly swollen from M to MA, and decomposed and melted in LA (Figure 2L). Similarly, the pyknosis of the cell nucleus was observed in LA (Figure 2P).

### 2.3. RNA-seq Analysis and Identification of Common DEGs

Twelve sample libraries were sequenced in total. As shown in Table 2, the clean reads ranged from 55.18 to 61.53 Mb in different treatment, and the Q30 percentage of all treatments was above 93%, which demonstrated the high quality of the sequence data. The percent of total mapped reads increased from 76.93 to 94.77%, and it was notable that the number of total mapped reads and clean reads decreased with the procedure of premature senescence.

**Figure 3:**
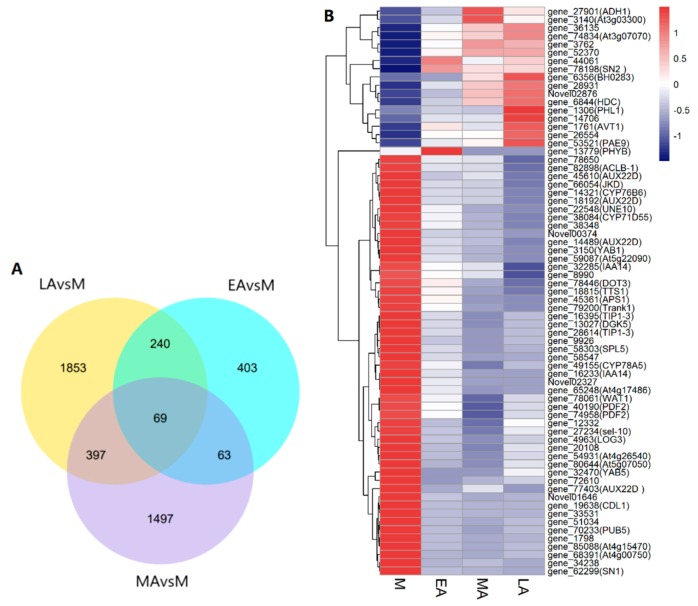
Venn diagram of differentially expressed genes (DEGs) and heat map of the 69 common DEGs. (**A**) Venn diagram of DEGs at three premature senescence stages compared with the mature stage. (**B**) Expression patterns of 69 common DEGs in the process of premature senescence. The *x*-axis represents each stage of premature senescence. The *y*-axis refers to gene expression levels. Log_10_(FPKM + 1) was used for the heat map to normalize the gene expression level. The gradient from blue to red indicates that the relative levels of gene expression range from low to high. FPKM, expected number of Fragments Per Kilobase of transcript sequence per Millions base pairs sequenced.

The differentially expressed genes (DEGs) at four senescence stage were investigated to identify the genes associated with premature senescence (Figure 3A, Table 3). A total of 775 DEGs were obtained between EA and M treatments, out of which 241 were up-regulated and 534 were down-regulated in EA. As for the comparison between MA and M, 2056 genes were found to be DEGs including 1032 up-regulated genes and 994 down-regulated genes. Furthermore, there were more DEGs in LAvsM than the other two comparisons, with 878 genes up regulating and 1681 genes down regulating, indicating that the difference compared with mature leaves increased with senescence stages. The three degrees of premature senescence (EA, MA, and LA) shared 69 common DEGs, related to senescence and expression patterns needing to be explored and discussed further. Thus a heat map for the 69 DEGs was generated (Figure 3B).

### 2.4. Functional Classification of Common DEG

**Figure 4:**
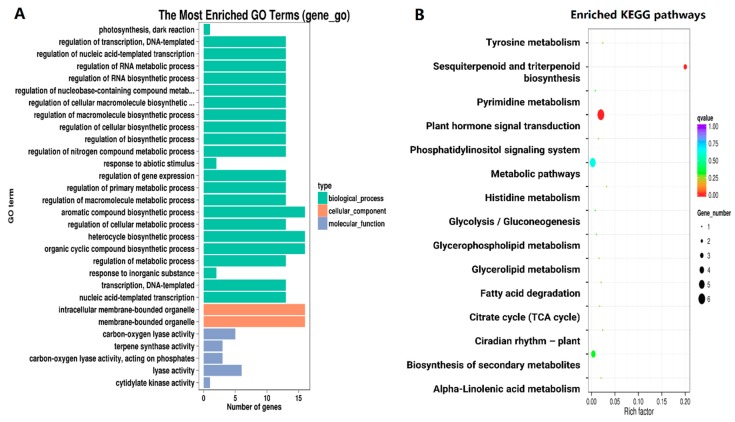
Gene Ontology (GO) and the Kyoto Encyclopedia of Genes and Genomes (KEGG) analysis of common DEGs in the process of premature senescence. (**A**) Barplot of significantly enriched GO terms. The *x*-axis represents the number of genes belonging to the left GO terms. The *y*-axis indicates GO terms. (**B**) Enriched KEGG pathways. Rich factor represents the ratio of the number of enriched DEGs in the KEGG pathway and the number of annotated background genes in this pathway. The *x*-axis shows rich factor of each pathway. The *y*-axis refers to KEGG pathways.

The significantly enriched GO terms of common DEGs from different stages of senescence were analyzed. Among these common DEGs, 51 genes were enriched in a number of terms. We selected the most significant terms to preliminarily cluster the function of these DEGs, as shown in Figure 4A. In the cellular component, intracellular membrane-bounded organelle and membrane-bounded organelle were overrepresented, both with the most gene number. The majority of terms were enriched in the biological process, including the aromatic compound biosynthetic process, heterocycle biosynthetic process, organic cyclic compound biosynthetic process, the regulation of cellular component synthesis, such as DNA-template, nucleic acid-template, RNA, cellular macromolecule and so on, as well as the regulation of other metabolic and biosynthetic processes. For the molecular function, the lyase activity, terpene synthase activity, and cytidylate kinase activity were mainly overrepresented, whereas few genes were enriched.

A total of 69 common DEGs was analyzed to further investigate the metabolic function, in which 15 KEGG (Kyoto Encyclopedia of Genes and Genomes) pathways were enriched and two pathways (sesquiterpenoid and triterpenoid biosynthesis and plant hormone signal transduction) were significantly enriched (Figure 4B). These pathways mainly related to the primary metabolism, including fatty acid degradation, alpha-linolenic acid metabolism, citrate cycle, glycerolipid metabolism, glycerophospholipid metabolism, pyrimidine metabolism, and glycolysis/gluconeogenesis. Also some pathways belonged to nitrogen metabolism, such as histidine metabolism and tyrosine metabolism. The rest of the pathways were correlative to secondary metabolism and plant hormone signal transduction. 

### 2.5. Expression Patterns of DEGs in Different Pathways Related to Senescence

**Figure 5:**
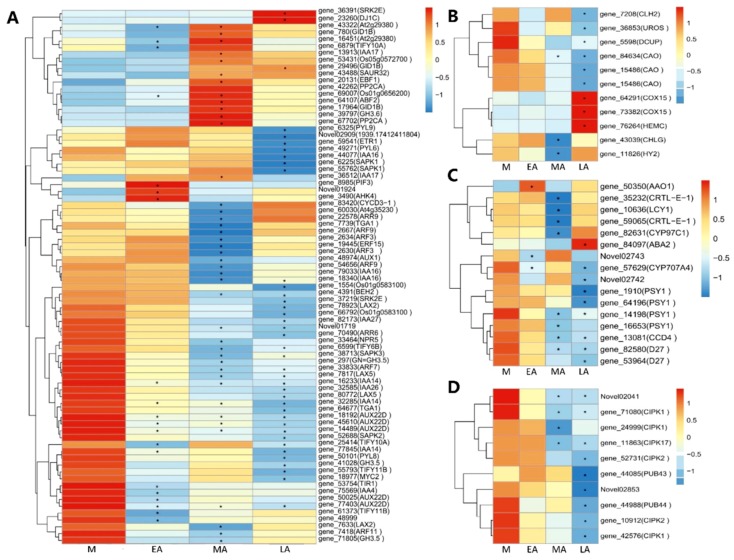
Expression patterns of DEGs in different KEGG pathways. (**A**) Plant hormone signal transduction. (**B**) Porphyrin and chlorophyll metabolism. (**C**) Carotenoid biosynthesis. (**D**) Regulation of autophagy. * represents a significant difference of gene expression levels at *p* ≤ 0.05 compared with M. The *x*-axis refers to each stage of premature senescence. The *y*-axis indicates gene expression levels. Log_10_(FPKM + 1) was used for the heat map to normalize the gene expression level. Blue and red represent low and high gene relative expression levels respectively.

To explore the expression patterns during premature senescence, several enriched and significantly enriched pathways were chosen, including plant hormone signal transduction, porphyrin and chlorophyll metabolism, carotenoid biosynthesis, and regulation of autophagy, which included all the DEGs from the three comparisons associated with these pathways. Expression heatmaps are shown in Figure 5.

In the plant hormone signal transduction pathway (Figure 5A), 78 genes were enriched, consisting of 20, 44, and 39 genes significantly expressed in EA, MA, and LA respectively compared with M. Except for some irregular gene expressions, most genes expressed down-regulated from M to LA, such as *BEH2*, *SRK2E*, *LAX2*, *IAA27*, *AUX22D* and so on, while a minority showed up-regulated during senescence, and of special concern were the *GID1B* and *SAUR32* genes.

A total of ten genes were enriched in porphyrin and chlorophyll metabolism pathway (Figure 5B), three genes significantly expressed in MA, and eight genes in LA compared with M. There were some genes showing progressive expression profiles in this pathway, like *CAO*, *COX15,* and *DCUP*, rather than those possessing unfixed and inconclusive regulation

The carotenoid pathway involved 16 genes (Figure 5C), most of which were down-regulated during premature senescence, only one gene (*ABA2*) expressed the opposite high in LA. This can be more focused on and discussed.

Genes significantly enriched in regulation of autophagy pathway (Figure 5D), showed all to be down-regulated during premature senescence, demonstrating that the deficit of autophagy could result in early senescence [53]. Obviously, the significant genes mostly existed in leaves of LA, and a part of them in MA, which demonstrated the extent of senescence.

### 2.6. Confirmation of RNA-seq Expression Levels by qRT-PCR

**Figure 6:**
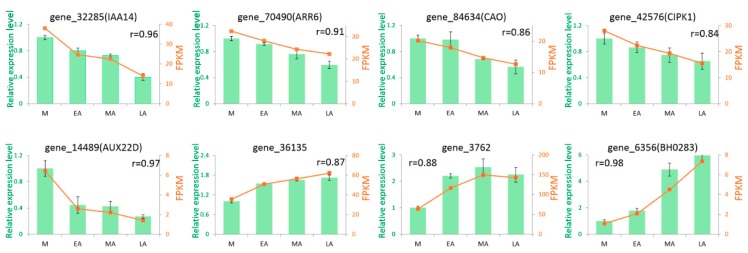
qRT-PCR validation of selected transcripts in tobacco leaves. The green columns represent the relative intensity of real-time quantitative RT-PCR (left *y*-axis), the red lines represent the FPKM value of the transcripts (right *y*-axis). The *x*-axis refers to the four stages of premature senescence. Error bars refer to standard error (n = 3). The values of r mean the Pearson correlation coefficient of data between RNA-seq and qRT-PCR.

To validate the gene expression patterns obtained from RNA-seq, qRT-PCR (real time quantitative reverse transcription polymerase chain reaction) was performed. Eight genes were chosen to detect the relative expression levels in different stages of premature senescence. Among them, gene_32285 and gene_70490 were related to the plant hormone signal transduction pathway. Gene_84634 was implicated in the porphyrin and chlorophyll metabolism pathway. Gene_42576 was associated with the regulation of autophagy. The other four genes belonged to the 69 common DEGs. The eight genes were all down-regulated or up-regulated with the process of premature senescence, which should be focused on. The results of qRT-PCR showed high accordance with the transcriptome determination, asthe eight genes possessed a high correlation coefficient (r > 0.84) between the two platforms (Figure 6). Thus, these results demonstrated that the transcriptional data from RNA-seq was credible and can be used for transcriptome analysis.

## 3. Discussion

Senescence is an age-dependent degradation process, most of which has been well studied in previous researches, but the mechanism associated with combined stress-induced senescence still remains unclear [31]. To avoid the damage from stress conditions, a strategy of accelerated-senescence could be performed in plants [54], so that the seeds of the plant are preserved but the yield and quality are reduced [31]. In this study, we choose four stages of senescence, to investigate the changes at cell ultrastructure and transcriptional levels. In the process of the mature stage (mainly during June and July), plants usually suffered relatively high temperature, high air humidity, and sometimes strong light intensity in Guangchang (Supplementary Table S6), which might induce premature senescence. It is well known that the content of MDA and chl have been used as an effective indication of leaf senescence [55]. The visible yellowing of leaves began here to deepen gradually because of the transformation from M to LA, with a correlated decline in chl content and an increase in MDA content (Figure 1), which demonstrated that premature senescence occurred in the tobacco leaves with the same genotype. 

For plant growth, a structural integrity needs to be maintained to provide photosynthesis [56]. In our study, with the process of aging, the phenomenon of plasmolysis appeared, and the damages to chloroplast, mitochondria, and cell nucleus became more serious. This was related to the metabolism of the substance and energy via organelles, verifying that the leaves selected at the same time point were indeed senescent rather than only temporary yellowing symptoms. Also, when the chloroplast structure was damaged, a decrease was observed in chlorophyll content (Figure 1B) that accelerated senescence [57]. The changes of starch grains were relative to the changes of chloroplast ultrastructure [58]. As shown in Figure 2, there was little difference in the quantity of chloroplast and starch grains between M and EA, but with the deepening of the aging, gradual decline and degradation were found in the starch grains and chloroplast, indicating that leaf senescence had commenced. As well the osmiophilic granules, the number of which increased during senescence, led to the damage and degradation of chloroplast [59]. A regular increase in osmiophilic granules from M to EA could be observed in this research, being similar to a previous report [60].

During leaf senescence, not only the phenotype, physiological activity, and cell structure, but the process are always accompanied by changes of a large number of genes [8], involving the anabolism and catabolism of carbohydrates, proteins, lipids and so on [17], as well as the signaling pathways of senescence [61,62]. At four stages, the number of down-regulated genes increased from M to LA, while the number of up-regulated genes reached the highest in MA, which probably demonstrates that the signaling mostly appears in the middle stage of senescence (Table 3). To explore the specific and precise genes associated with premature senescence, we chose the 69 common DEGs from three stages of aging leaves, further analyzing the categories and functions by GO and KEGG enrichment (Figure 4).

Through the GO enrichment analysis, we detected a total of 105 significant overrepresented terms. Organelles are often double or single membrane bounded, like mitochondria, peroxisome, and chloroplast. The genes in the term of membrane-bounded organelle and intracellular membrane-bounded organelle were down-regulated, in accordance with the result of ultrastructure. Also, except for the regulation of metabolic and biosynthetic process, regulation of cellular component synthesis accounted for a large proportion, speculating possible patterns in premature senescence. Focusing on the KEGG results, many of the 15 pathways were verified to be associated with senescence by previous reports, for example, circadian rhythm-plant and tyrosine metabolism were enriched in senescent sorghum [47], alpha-linolenic acid metabolism existed in Arabidopsis [63], and premature-senescent wheat mutant [64], which showed that the senescence or premature senescence was partly the same and partly distinctive.

Of these genes, the hormone-related genes were expressed significantly, including the regulation of IAA, ABA, JA, and BRs. PIN-LIKES (PILS), which was reported as a novel putative auxin transport facilitator family, could regulate the intracellular content of auxin [65]. Gene_36135, a protein PIN-LIKES 6-LIKE gene, was expressed low in M, but up-regulated gradually in the samples from EA to LA, which catalyzed cellular auxin efflux to reduce auxin accumulation. The expression of this gene was proved to be coincident in the premature senescence of *Gossypium hirsutum* [7] and was definitely focused. When the application of IAA was increased, the transcript level of AUX22D (auxin-induced protein 22D) increased likewise [66]. Four genes, encoding AUX22D, were down-regulated significantly in the three stages of senescence. Also, the genes modulating IAA14, known as the repressor of auxin signaling [67], were inactivated in the expression during senescence. All of these obviously revealed the reduced accumulation of IAA, indicating that the auxin signaling and transport were important in the regulation of senescence. In addition, the ABA and environmental stress-inducible protein TAS14-like were activated in the process of senescence, especially in the last two stages (Figure 3B). Previous findings indicated that the exogenous methyl jasmonate triggered an elevated expression of *SN2* gene which was involved in JA-dependent defense response [68], the expression of which increased significantly during senescence (Figure 3B). Whatis more, *CDL1*, a homolog of *CDG1*, which positively regulated brassinosteroid signaling and plant growth, was expressed negatively at the initiation and procedure of senescence. The molecular patterns of hormone-related genes may prove the positive roles of ABA, and JA, and the negative role of IAA to generate senescence. 

Some other genes were also up- or down-regulated significantly during senescence. ATP-related genes, such as *ACLB-1* and *APS1*, encoding ATP citrate lyase and ATP sulfurylase respectively, were reduced from the beginning of senescence. In accordance with the result from Pourtau, *APS1* was repressed by sugar-induced senescence [69]. A down-regulated gene, *CYP71D55*, was annotated functioning in gene expression, organogenesis, and meristem development. Mutation of *PEA9* leads to about 20% increase of acetate content and accommodates the acetylation state of pectic polymers in cell walls [70], and our study recognized an up-stream process in expression of *PAE9*, which indicated acetate and pectic acetylation may participate in the process of senescence. Some morphological and physiological changes could be found when plants were exposed to abiotic stresses, as well as the vein patterning, in which the genes that encode DOT3 play multiple important roles [71]. We identified a gene encoding DOT3 significantly declined from senescing, affecting venous tributaries.

According to the expression levels in the samples, we analyzed other genes which were relative to senescence but not functionally annotated or reported, such as gene_74834 (*At3g07070*), gene_6356 (*BH0283*), gene_6844 (*HDC*), gene_1306 (*PHL1*), gene_52370, gene_26554, gene_28931, Novel02876, Novel02327 and so on, indicating a further resource of genes may participate in premature senescence.

Not only the common DEGs, but also some DEGs significantly enriched in senescence-associated pathways, provided valuable reference for expression patterns and gene modulation. As a role of accelerating or delaying leaf senescence, plant hormone laid a big foundation for senescence [72]. As is well known, most genes associated with abscisic acid (ABA), ethylene, salicylic acid, brassinosteroid and jasmonic acid positively regulate senescence, however many auxin, cytokinin and gibberellin related genes do the opposite [7]. A large proportion of IAA-response family genes (*IAA4*, *IAA14*, *IAA16*, *IAA26* and so on), auxin-induced protein 22D genes (*AUX22D*), and auxin transporter-like protein genes (*LAX2* and *LAX5*) showed similar down-stream with the appearance of senescing, strongly demonstrating the reduction of auxin. However, the IAA1d oxidase, nitrilases and the auxin efflux carrier family genes were not significantly differently expressed [73]. *ARR6* encodes a Type-A response regulator that is responsive to cytokinin treatment, being reduced in premature senescence. The homologs of GA receptor *GID1* [74] were almost up-regulated when premature senescing, however the trends of which were opposite in premature senescence cotton [7]. The ABA-induced genes, *PP2C*, increased in their mRNA levels significantly from the phase of MA as we discovered, which could appear to dephosphorylate an essential component of the ABA pathway leading to the synthesis of hydrolytic enzymes and programmed cell death (PCD) [75]. This mediation proved coincident in petunia corolla senescence [49]. Whereas the three genes encoding PYL family proteins which act as abscisic acid sensors, were obviously down-regulated in LA compared with M, maybe demonstrating the transcriptional reduction of PYL homologs in extreme premature senescence. In addition, the homolog 2 of *BES1*/*BZR1*, *BEH2*, was down-regulated significantly in MA and LA, The BRs signaling positive regulator showed reduced transcription levels with processive premature senescence, as well as the expression patterns of *CDL1* (Figure 5A). In the latest research, the transcript factor *TGA1* positively regulates SA biosynthesis by modulating the expression of target *SARD1* [76], to effect plant immunity [77]. The expression of *TGA1* decreased from M to LA, markedly in MA or LA.

These results seem to indicate the complexity of the unique hormone regulation mechanism for premature senescence, not only in the overall variety of each hormone, but also the unique patterns and interactive cross-talk between every gene. The most obvious trend belonged to down regulation, of which it could be deduced that such regulation could be more regular and function less in senescence.

The porphyrin and chlorophyll metabolism undergoes a process from being catalyzed by l-glutamate and synthesis of chl precursors to the formation of chl a/b [78]. CAO is well accepted as chlorophyll a oxygenase, catalyzing chl a to chl b [79]. With progressive premature senescence, two genes encoding CAO were downregulated, demonstrating the decrease of chl b and signaling a decrease of chl a. The sudden rise at the last senescence period in the transcriptional levels of *COX15* homologs, which act on the synthesis of heme A from heme B along with *COX10* and as electron acceptor of the respiratory chain [80], implied its possible role in plant senescence [81], but it still remains elusive. The expression of uroporphyrinogen decarboxylase (DCUP) could be repressed under 16 d of N starvation [82], which was the key regulatory enzyme for synthesis of chlorophyll. Significant down-regulation in LA was found in our study. 

Carotenoid biosynthesis pathway shares an overlapping expression pattern of genes with ABA biosynthesis, involving *ABA2*, *AAO3,* and *NCED3*, and ABA positively modulates the carotenoid biosynthesis in turn [83]. In our study, the only up-regulated gene *AAO3*, was described as a NAD(P)-binding Rossmann-fold superfamily protein gene, with the characteristic of encoding a dehydrogenase/reductase to catalyze xanthoxin to ABA precursor [84]. The upregulation of ABA2 in LA confirmed the increase of carotenoids and ABA. Additionally, *PSY1* was also reported relative to ABA biosynthesis [36]. However, the expression profiles of *PSY1*, *CCD4,* and *D27* all exhibited down regulations, revealing the diversity of the molecular network between proteins and metabolites.

Under most circumstances, the adaption to stress conditions for cells can be promoted by autophagy [85]. Most studies support the findings that cell death will be accelerated by apoptosis after the functional blocking of autophagy [86] and autophagy negatively regulates early senescence and excessive immunity-related PCD [53]. In the last stage of premature senescence, most genes were down-regulated. This result was exactly coincident with previous research, revealing the possible role of autophagy in premature senescence.

In conclusion, this work used the investigation of phenotype, physiology, as well as cell and gene levels to verify the changes in premature senescence. The degradation of organelle was confirmed; and also potential genes that signal, and modulate premature senescence were identified. The possible dynamic network of gene expression patterns identified, provided information to understand tobacco premature senescence.

## 4. Materials and Methods

### 4.1. Plant Materials and Growth Condition

*Nicotiana tabacum* cv. Yunyan 87 was used in this study, which was widely cultivated in south China. Field experiments were carried out at the experimental station in Guangchang, Jiangxi province, China (Lat 26°33′ N, Long 116°53′ E). Leaves of premature senescence were observed in Guangchang for a consecutive two years, then we carried out the field experiment on the same ground, based on the controlled variables of basic soil fertility, fertilization, watering and so on. The seeds were sterilized with 2% sodium hypochlorite for 10 min, and sown in a plastic pot on the 15th January 2017, then transplanted into soil after 25 days. The basic soil fertility consisted of 18.37 g/kg organic matter, 53.64 mg/kg available nitrogen, 8.40 g/kg and 42.83 g/kg rapid available phosphorus and potassium respectively, while 8.72 g, 9.02 g, and 27.64 g pure nitrogen, phosphorus and potassium were added per plant. Three years of main meterological conditions of the experiment site were monitored, including the light radiation intensity, daily maximum temperature, and air humidity (Supplementary Table S6). The plants were planted at row spacing of 1.2 m and plant spacing of 0.5 m, and grown under the same local cultivation conditions.

### 4.2. Plant Materials Used in RNA-Seq

Samples of leaves were selected on 23 June, when the fifteen leaves (counted from the bottom to the top) were mostly mature but not senescent and others were senescent. We chose the leaves of four stages at the same time, which were defined as mature (M), early premature senescence (EA), middle premature senescence (MA), and late premature senescence (LA). The degree of senescence was judged by visible symptom leaf yellowing rate at first [50,54,55] and then verified by physiological measurement and ultrastructure analysis. Three biological replicates were used for RNA-Seq and physiological measurement; the samples of each biological replicates were pooled from 3 plants, to avoid the potential effects of position and nutrition.

### 4.3. Chlorophyll and MDA Content Measurement

Chlorophyll (chl) was extracted using 80% (*v*/*v*) acetone, ground into homogenate, and then stored in darkness for 10 min. The samples were centrifuged at 12,500× *g* for 10 min, and the absorbance of supernatant was measured at 470, 646, and 663 nm with a spectrophotometer (UV-1780, Shimadzu, Japan). The calculation of chlorophyll was as described by Liu [87]. MDA contents were measured using 5% thiobarbituric acid, as described by the method of Saher [88].

### 4.4. Transmission Electron Microscopy Analysis

Sections of leaves (1 mm × 2 mm) were cut and immediately put into 4% glutaraldehyde which was cooled down to 4 °C, then vacuumed to make the leaves sink. After two days’ store at 4 °C, samples were fixated with 1% OsO4, dehydrated by acetone, and then embedded with epoxy resin (Epon-812). After being stained with uranyl acetate and lead citrate, the ultrathin sections cut by LKB were viewed and photographed with an electron microscope (Hitachi 600, Tokyo, Japan) [89].

### 4.5. RNA Extraction, Library Construction and Illumina Sequencing 

A total amount of 1 µg RNA per sample was used. Libraries were generated using NEBNext® Ultra™ RNA Library Prep Kit for Illumina® (NEB, Beverly, MA, USA) according to the manufacturer’s recommendations, then they were sequenced on an Illumina Hiseq platform and 125 bp/150 bp paired-end reads were generated. Row reads were purified to obtain clean reads, then were mapped to the tobacco reference genome (ftp://anonymous@ftp.solgenomics.net/genomes/Nicotiana_tabacum/assembly/Ntab-K326_AWOJ-SS.fa.gz).

### 4.6. Transcriptome Analysis

FPKM, expected number of Fragments Per Kilobase of transcript sequence per Millions base pairs sequenced, is currently the most commonly used method to evaluate gene expression levels [90]. Differential expression analysis of two groups (three biological replicates per group) was performed using the DESeq R package (1.18.0, European Molecular Biology Laboratory, Heidelberg, Germany) [91]. The resulting *p*-values were adjusted using the Benjamini and Hochberg’s approach for controlling the false discovery rate. Genes with *p*-values ≤ 0.05 found by DESeq were assigned as differentially expressed genes (DEGs). Heat map was performed by the pheatmap R package. Data for the heat map was normalized by log_10_(FPKM + 1) and standardized by z-score to meet the standard normal distribution. Gene Ontology (GO) enrichment analysis was implemented by GOseq R package. GO terms with *p*-values ≤ 0.05 were considered as significantly enriched terms. KOBAS software (2.0, Center for Bioinformatics, Peking University, Beijing, China) [92] was used to test the statistical enrichment of DEGs in Kyoto Encyclopedia of Genes and Genomes (KEGG) pathways, and pathways with *q*-values (corrected *p*-values) ≤ 0.05 were regarded as significantly enriched pathways.

### 4.7. Real-Time Quantitative RT-PCR

To validate the results of RNA-data, expressions of 10 genes which were selected randomly were measured by qRT-PCR to test accordance with RNA-seq, both RNA-seq, and PCR while sharing the same samples. The reactions of reverse transcription (RT) and PCR were performed using a GeneAmp® PCR System 9700 (Thermo Fisher Scientific Applied Biosystems, Carlsbad, CA, USA) and LightCycler® 480 II Real-time PCR Instrument (Roche, Basel, Swiss) respectively. PCR reactions were incubated at 95 °C for 5 min, and 40 cycles of 95 °C for 10 s, 60 °C for 30 s, using a 384-well optical plate (Roche, Basel, Swiss). Then the melting curve analysis was carried out to confirm the specific generation of the expected PCR product. According to the mRNA sequences obtained from NCBI (Supplementary Table S1), the primers were designed and synthesized by Generay Biotech (Shanghai, China). The expression levels were normalized by the gene L25 as an internal control and were evaluated according to previous study [93].

## Figures and Tables

**Table 1:** Ultrastructural changes in mesophyll cells during the process of premature senescence.

The Object of Observation	Treatments			
	M	EA	MA	LA
Plasmolysis	No	No	Slight	Serious
Mitochondria	Rounded	Rounded, flattened	swelling, densified matrix contains crista	degraded, broken
Cell Nucleus	Rounded	Compressed	Compressed, pyknotic	Broken, pyknotic
Chloroplast	Stacked, fat	Stacked	Scattered, wizened	Most disintegrated
Starch grains Area ^a^ (%)	50–70	40–70	10–20	<5, rarely in chloroplasts
Osmiophilic granules Size (μm)	0.02–0.5	0.1–1.0	0.1–1.2	0.1–1.4
Number ^b^	3–8	3–11	3–13	12–18
Thylakoid lamellae	arranged in order	arranged in order	arranged in order, but expanded slightly	expanded seriously

^a^ Starch grans area per chloroplast area. ^b^ Number of osmiophilic granules in chloroplast and cytoplasm. M: Mature; EA: Early Senescence; MA: Middle Senescence; LA: Late Senescence.

**Table 2:** Summary of sequencing data.

Treatments	Clean Reads (Mb)	Data Size (G)	Q20 (%)	Q30 (%)	GC Content (%)	Total Mapped Reads (%)	Multiple Mapped Reads (%)	Uniquely Mapped Reads (%)	Reads Mapped to Exon (%)	Reads Mapped to Intergenic Regions (%)
M	61.50	9.22	97.51	93.69	42.99	94.77	2.38	92.39	90.27	7.57
EA	61.53	9.23	97.32	93.32	42.75	85.94	2.18	83.76	89.17	8.47
MA	60.93	9.14	97.49	93.63	42.80	83.48	2.13	81.35	89.60	8.23
LA	55.18	8.23	97.56	93.79	42.75	76.93	1.90	75.03	89.90	7.90

**Table 3:** Differentially expressed genes (DEGs) in different stage of premature senescence compared with M.

	DEGs	Up	Down
EA vs. M	775	241	534
MA vs. M	2026	1032	994
LA vs. M	2559	878	1681
common	69	17	51

## Supplementary Materials

The following are available online. Table S1: Primer sequences used for qRT-PCR expression analysis, Table S2: Common DEGs and their FPKM, Table S3: Top 30 enriched GO terms of common DEGs, Table S4: Enriched senescence-related KEGG pathways, Table S5: genes’ FPKM associated with four pathways, Table S6: Meteorological data.
